# Ameliorating transport-related stress in endangered Kemp’s ridley sea turtles (*Lepidochelys kempii*) with a recovery period in saltwater pools

**DOI:** 10.1093/conphys/coy065

**Published:** 2019-01-02

**Authors:** Kathleen E Hunt, Charles Innis, Constance Merigo, Elizabeth A Burgess, Terry Norton, Deborah Davis, Adam E Kennedy, C Loren Buck

**Affiliations:** 1 Department of Biological Sciences, Northern Arizona University, 617 S. Beaver St., Flagstaff, AZ, USA; 2 Animal Health Department, Central Wharf, New England Aquarium, Boston, MA, USA; 3 Rescue and Rehabilitation Department, Central Wharf, New England Aquarium, Boston, MA, USA; 4 Anderson Cabot Center for Ocean Life, Central Wharf, New England Aquarium, Boston, MA, USA; 5 Georgia Sea Turtle Center/Jekyll Island Authority, 214 Stable Road, Jekyll Island, GA, USA; 6 Idexx Laboratories, 3 Centennial Drive, North Grafton, MA, USA

**Keywords:** Chelonian, corticosterone, glucose, soft release, stress, transportation

## Abstract

Sea turtle rehabilitation clinics and aquaria frequently transport stranded sea turtles long distances out of water, e.g. for release at sites with appropriate water temperatures. Endangered Kemp’s ridley turtles (*Lepidochelys kempii*) are known to exhibit an adrenal stress response during such transports. In an opportunistic study of turtles transported by road from Massachusetts to Georgia for release, we tested whether placing turtles in saltwater pools for short periods after transport would help turtles recover from transport-related stress. Eighteen juvenile Kemp’s ridley turtles were examined and blood samples collected (1) immediately pre-transport, (2) immediately post-transport and (3) after a 6 h (*n* = 9) or 24 h (*n* = 9) post-transport period in unfamiliar pools, after which all turtles were released to the sea. Blood samples were analyzed for corticosterone, glucose, total white blood cell (WBC) count, heterophil/lymphocyte (H/L) ratio, pH, pO_2_, pCO_2_, HCO_3_ (bicarbonate), sodium, potassium, ionized calcium, lactate and hematocrit. Though the majority of turtles remained in good clinical condition, corticosterone, glucose, WBC and H/L elevated significantly during transport, while potassium declined slightly but significantly. After at least 6 h in a saltwater pool, potassium and glucose returned to pre-transport baselines and corticosterone partially recovered toward baseline. Extending the pool time to 24 h did not markedly enhance the physiological recovery of turtles, and two immune measures (WBC, H/L) remained elevated from the effect of transport. Six hours in a saltwater pool appears to facilitate the recovery of Kemp’s ridley sea turtles from transport-related stress and may therefore improve their readiness for release.

## Teaser

Kemp’s ridley sea turtles placed for <6 h in unfamiliar saltwater pools after ground transport exhibited normalization of plasma glucose and potassium, as well as partial improvements in corticosterone and immune measures. A brief period in saltwater pools may help sea turtles recover from transport stress prior to release to sea.

## Introduction

During the past decade, increasing numbers of juvenile Kemp’s ridley sea turtles (*Lepidochelys kempii*, ‘Kemp’s ridley turtles’), a critically endangered species, have been found stranded during fall on the shores of Cape Cod, MA, USA (Allman, 1998; Gerle *et al.*, 2000; Still *et al.*, 2005; Innis *et al.*, 2014; Rockwell *et al.*, 2017; Spielvogel *et al.*, 2017; NOAA, 2018). The stranded turtles are typically ‘cold-stunned,’ i.e. in a hypothermia-like state that involves reduced body temperatures and related clinical abnormalities, including dehydration, electrolyte derangements, acidosis, pneumonia, sepsis and traumatic injuries (Innis *et al.*, 2009; Innis and Staggs, 2017). The majority of cold-stunned Kemp’s ridley turtles are rehabilitated successfully (Innis and Merigo, 2014) and are ready for release to the sea within a few months (often by March of the following year), but at that time local water temperatures are too low for safe release of these ectothermic, migratory animals. To expedite their return to the wild, the turtles must be transported from the initial rescue site (Massachusetts) hundreds of miles south for release at beaches with appropriate water temperatures, e.g. to southern U.S. states such as Georgia and Florida. Since 2013, several hundred threatened sea turtles each year have required transportation south along the U.S. eastern seaboard to seasonally appropriate release sites (NOAA, 2018). The large majority of transported turtles are Kemp’s ridley turtles, with occasional loggerhead turtles, *Caretta caretta*, and green turtles, *Chelonia mydas*. This annual flow of rescued and rehabilitated sea turtles southbound along the U.S. eastern seaboard during spring has become a regular wildlife transportation event in North America.

As sea turtles are air-breathing vertebrates, for logistical reasons these transportation events (‘transports’) typically occur with the turtles out of water in individual padded crates or boxes. Ground transports in trucks or vans are the most common method of transport; aircraft transports also occur, but are a minority of transports. Even with streamlined protocols—overnight driving shifts with multiple teams of drivers, minimal stops for refueling—ground transports regularly exceed durations of 12 h and sometimes approach 36 h. Pre-transport preparation (e.g. veterinary exams, loading turtles into crates) and post-transport delays at the release site (e.g. unloading of crates from the vehicles, delays associated with beach logistics and public or media relations) can add additional hours to the total out-of-water period experienced by the turtles. For clinical and management decisions, it is therefore of interest to determine the degree of physiologic stress that sea turtles might experience during prolonged transport events and to evaluate whether modification of existing practices could reduce any such stress before release.

Generally, transportation of wildlife involves exposure to a suite of unfamiliar stimuli including confinement in a small and novel container (i.e. a crate or box), vehicle noise, vibration, unpredictable acceleration and, in the case of sea turtles, lack of access to food and a significant period of time removed from their aquatic environment (Grandin, 2007). Most vertebrates respond to transport-related stressors with a physiological response characterized by a several-fold increase in adrenal hormones (corticosterone, cortisol and catecholamines; Grandin, 2007; Knowles and Warriss, 2007). These hormones coordinate a systemic ‘stress response’ that increases fuel availability (e.g. gluconeogenesis and lipolysis), alters behavior (e.g. increased foraging, abandonment of familiar areas and increased spatial movement) and temporarily suppresses nonessential processes such as reproduction and growth (Sapolsky *et al.*, 2000; Romero and Wingfield, 2015). The stress response also redistributes immune cells between vascular and interstitial compartments, often detectable via increases in blood heterophil/lymphocyte (H/L) ratio and increases in total white blood cell (WBC) count (Martin, 2009; Romero and Wingfield, 2015). In the short term (minutes to hours), this physiological stress response is adaptive, serving to redirect available energy toward rectifying metabolic or other imbalances due to exposure to the stressor (Romero and Wingfield, 2015). However, prolonged or repeated stress responses have negative long-term effects including suppression of growth in juveniles, increased vulnerability to disease, increased wound-healing time, impaired reproduction, decreased ability to mount a new stress response for any new stressors and long-term alterations in behavior (reviewed in Romero and Wingfield, 2015). In wildlife released to the wild immediately after transportation (‘hard release’), immediate impacts can include an increased tendency to abandon the release site, increased vulnerability to predators and disease and concomitant increased risk of mortality (Jenni *et al.*, 2015). Long-term effects of stress can last months to years, including persistent effects on growth, adult body size, adult behavior and adult reproduction (Romero and Wingfield, 2015). Though such impacts of stress have not been assessed in chelonians, these patterns occur in almost all vertebrate taxa that have been studied (Romero and Wingfield, 2015), suggesting caution when exposing wildlife to stress. Therefore, though some stress is clearly inevitable when transporting wildlife, it has been recommended that transport practices be adjusted to minimize and/or ameliorate stress where feasible, especially those transports that occur immediately prior to release (Grandin, 2007; Teixeira *et al.*, 2007; Dickens *et al.*, 2010; Jenni *et al.*, 2015).

In a prior study, we confirmed that Kemp’s ridley turtles experience an adrenal stress response during ground transports of 13 and 26 h durations, with pronounced and significant elevations in plasma corticosterone, plasma glucose, WBC and H/L ratio immediately post-transport as compared to pre-transport (Hunt *et al.*, 2016a). Controlled experiments clarified that these changes were primarily due to the transportation event, with only minor effects attributable to handling or time-of-day (Hunt *et al.*, 2016a). Such changes do not necessarily indicate that turtles are adversely affected from a clinical standpoint—in fact, vital rates typically remained within normal ranges, as did other clinical measures such as pH, blood gases and electrolytes (Hunt *et al.*, 2016a). However, given the potential short- and long-term impacts described earlier, it is worth exploring methods to ameliorate transport-related stress in sea turtles. An increasingly common management option employed with other species is ‘soft release,’ holding animals in the vicinity of the release site for some period of time just after transport and before release into the wild (Jenni *et al.*, 2015). For sea turtles, soft releases could involve placement in saltwater tanks or pools near the release site, which, although not fully mimicking conditions in the wild, might at least ameliorate some of the physiological stress caused by transportation. The pool would itself be an unfamiliar environment for the turtles however, so it has been unclear whether such an approach, could cause more stress than it alleviates.

In 2015, an opportunity arose to examine whether the physiological stress response caused by overnight transport of Kemp’s ridley turtles could be ameliorated by placing turtles temporarily in a saltwater pool. Eighteen juvenile Kemp’s ridley turtles were scheduled for transport from Massachusetts to Georgia for release, and a sea turtle clinic near the release site had multiple saltwater tanks operational and available for testing of a soft release protocol. Given logistical factors of long-distance sea turtle transports from the New England region, two durations of soft release are most likely to be feasible: ~6 h in a pool (place turtles in pool on arrival in the morning, release at beach that afternoon), or ~24 h in a pool (place turtles in pool on arrival in the morning, release at beach the following morning). We tested these two approaches by comparing physiological parameters of turtles before transport, immediately post-transport and after either ~6 or ~24 h in a saltwater pool. Specific questions were as follows: (1) Does a period in a saltwater pool cause significant changes in any of the four measures known to elevate during transport stress in Kemp’s ridley turtles (corticosterone, glucose, WBC, H/L ratio; Hunt *et al.*, 2016a), or in any of the other clinical measures that are routinely monitored (e.g. pH, blood gases, electrolytes, lactate, hematocrit)? (2) If so, does the unfamiliar pool ameliorate stress or worsen it? (3) If placement in a pool ameliorates physiologic stress, do stress-related measures return fully to pre-transport baseline levels? (4) Is increased time in the pool (24 h as compared to 6 h) associated with continued improvements in stress-related measures?

## Materials and methods

### Study subjects

Our experimental design involved the opportunistic study of eighteen juvenile Kemp’s ridley turtles that were scheduled for transport from the New England Aquarium’s sea turtle rehabilitation center (‘NEAq’, Quincy, MA, USA) to the coast of Georgia for release. All turtles had stranded in a cold-stunned state on the northern shore of Cape Cod during the previous fall (admission dates of October–December 2014). All turtles were estimated to be 2–3 years of age based on carapace length at the time of stranding compared to known-age conspecifics (B. Higgins, National Marine Fisheries Service, pers. comm.; Avens and Snover, 2013).

Our clinical treatment of cold-stunned Kemp’s ridley turtles has been fully described elsewhere (Wyneken *et al.*, 2006; Innis and Staggs, 2017). Briefly, turtles were gradually rewarmed during their first week of hospitalization while medical problems were treated. Once swimming well, turtles were placed in large tanks with other similar-sized turtles, with individualized veterinary treatment continued as necessary. Turtles were subsequently maintained in saltwater tanks at ~26°C (the average of the preferred temperature range for this species; Renaud, 1995) as described in Hunt *et al.* (2012), with daily health monitoring by clinical staff and twice-daily feeding (herring and squid offered individually to each turtle). Behavior and corticosterone levels of cold-stunned Kemp’s ridley turtles return to presumed-normal levels after ~2 months in the NEAq rehabilitation clinic (Hunt *et al.*, 2012); turtles in this study had been housed in the NEAq clinic for 6–8 months before transport. Prior to this study, all turtles were judged by veterinary staff to be clinically stable and in good condition based on serial physical examinations, hematologic and plasma biochemical analysis and radiographic evaluation.

The week before transport, turtles were weighed and measured for straight carapace length (notch-to-tip) and straight carapace width (at widest point). Turtles were randomly assigned to one of two different pool groups, a ‘6 h’ group that would remain in the pools for ~6 h post-transport and a ‘24 h’ group that would remain in the pools overnight for an uninterrupted post-transport recovery period of ~24 h. (The 24 h group was not also sampled at 6 h due to the fact that such sampling would have interrupted the desired recovery period with a known stressor.) There were no obvious differences between experimental groups in clinical status, behavior or any other parameters. Turtles assigned to the two experimental groups were examined and sampled in alternating order during the pre-transport and immediately post-transport sampling events.

The NEAq sea turtle rehabilitation program and associated research were conducted with the authorization of the United States Department of the Interior Fish and Wildlife Service (permit number TE-697823), in compliance with guidelines of the Animal Care and Use Committee of the New England Aquarium (IACUC protocols 2012-03 and 2015-10). Turtle transports and beach releases were performed in compliance with all applicable local, state and federal regulations and guidelines.

### Sampling, handling and transportation

#### Sample 1 (pre-transport)

On the morning of 15 May 2015 immediately prior to transport, turtles were removed from their pools at the NEAq sea turtle rehabilitation clinic in Quincy, MA. Animals were gently scooped from the water with a hand-held net, slightly tipped for ~5 s to allow water to drain from the mouth, placed in a transport container and carried to an examination room. This process took <1 min. A 2–3 ml heparinized blood sample was immediately collected from the external jugular vein as previously described (Innis *et al.*, 2007, 2009), followed by measurements of cloacal temperature, heart rate and respiratory rate. Since handling and sampling can themselves cause stress responses (Romero and Reed, 2005; Hunt *et al.*, 2016a), blood sampling time (‘bleed time’) and total handling time were both timed from ‘time zero,’ defined as the time that the capture net first entered the turtle’s pool. Bleed time was generally <3 min and handling time <10 min (see Results); our prior studies indicate that these sampling and handling protocols have only minimal effects on the physiological measures presented here (Hunt *et al.*, 2016a). After handling was complete, turtles were placed in individual padded crates with air holes and prepared for overnight ground transport to Georgia.

#### Transportation protocol

Just prior to departure, each turtle received subcutaneous fluid therapy (lactated Ringer’s solution, 10 ml/kg). Each turtle’s skin and shell were manually coated with a thin layer of water-soluble lubricant to reduce dehydration. Crates were then closed and loaded into the rear cargo area of large sport utility vehicles, each vehicle also containing three personnel and equipment. Ambient temperature of vehicles was set to 26°C, monitored continuously with digital remote temperature probes placed in the rear cargo area near the turtles. Vehicles departed Quincy, MA on 15 May 2015 at 12:46 pm, drove throughout the night and arrived at the Georgia Sea Turtle Center (‘GSTC’) (Jekyll Island, GA, USA), the following morning at 9:24 am. Brief stops (~15 min) for refueling and driver-change occurred approximately every 3–4 h.

#### Sample 2 (immediately post-transport)

Upon arrival at GSTC, turtles were unloaded from vehicles and carried (still in closed crates) into the GSTC’s veterinary exam room, where the crates were opened. A second heparinized blood sample was taken (Sample 2), and a second clinical exam performed using the methods described above. Total duration of the ground transport event for each individual (including pre-transport preparation time and sampling time), averaged 23.78 ± 0.07 h (mean ± SD). After sampling, turtles were placed into seven saltwater pools at 11:00 am on 16 May 2015. Turtles were placed in their designated pools in groups of two or three. All turtles were placed with familiar turtles with whom they had shared housing during the prior months of rehabilitation at NEAq, and the two experimental groups (6 and 24 h pool times) were evenly divided within and across pools so as to minimize any influences of pool variation. Temperatures of the GSTC pools ranged between 24.5 and 25.1°C, comparable to (but slightly lower than) the ~26°C tank temperatures at NEAq. Turtles were not fed while in the GSTC pools.

#### Sample 3 (after pool)

After the prescribed time in the pool (6 or 24 h), turtles were examined and sampled a final time (Sample 3) with the protocols described above and were released to sea either on the evening of 16 May 2015 (6 h pool group) or the morning of 17 May 2015 (24 h pool group). Mean total time in pool (±SD) between Sample 2 and Sample 3 was 6.09 ± 0.02 h for the 6 h pool group and 24.18 ± 0.09 h for the 24 h pool group. All turtles appeared in good condition at the time of release, crawled vigorously when placed on sand, and swam well upon reaching the sea. Note that we have no post-release data from turtles in this study; they were not fitted with satellite tags because satellite tag attachment is itself a stressor known to affect corticosterone (Hunt *et al.*, unpub. data). Similarly, recapture of turtles for additional blood sampling was not possible, due to the lack of satellite tags as well as additional logistical, budgetary and permitting constraints. Parallel and ongoing studies will focus on post-release movement and survival of turtles in other releases.

### Blood analyses: i-STATs, complete blood counts and corticosterone assays

Whole-blood samples were divided into three portions as in Hunt *et al.* (2016a). A total of 0.20 ml was used for point-of-care i-STAT clinical chemistry analyses of electrolytes, glucose, lactate, blood gases and pH (see next section), and ~0.5–1.0 ml of whole blood was shipped on ice (not frozen) to an outside laboratory (IDEXX Reference Laboratories, North Grafton, MA, USA) for a complete blood count (CBC). The remaining blood was refrigerated until all turtles had been sampled (~45–60 min) and then centrifuged at 1500 × *g* for 5 min, with plasma pipetted to a vaporproof cryovial and shipped on dry ice to NEAq for temporary storage. The latter plasma samples were later shipped on dry ice to the endocrine laboratory of the Buck Laboratory, Northern Arizona University (Flagstaff, AZ, USA), for corticosterone assay (details below).

#### Clinical chemistry (i-STAT analyses)

Immediately upon collection of the blood sample, whole blood was loaded directly into both an i-STAT CG4+ Test Cartridge and to an i-STAT CG8+ Test Cartridge, which were analyzed using a portable battery-powered hand-held point-of-care analyzer, VetScan i-STAT®1 Analyzer Model 300 A (Abbott Point Of Care, Princeton, NJ, USA). Two i-STAT machines were used; each turtle’s three samples were analyzed by the same i-STAT machine. Generally, data appeared comparable across machines (data not shown). The CG4+ cartridges measured plasma pH, pO_2_, pCO_2_, HCO_3_ and lactate. The CG8+ cartridges measured sodium, potassium, ionized calcium (iCa) and glucose and also produced backup data on pH, pO_2_, pCO_2_ and HCO_3_. Data for pH, pO_2_, pCO_2_ and HCO_3_ were taken from the first cartridge analyzed, which in almost all cases was the CG4+ cartridge. In five cases (three turtles from the 6 h pool group and two from the 24 h pool group), the CG4+ cartridge initially failed and hence the CG8+ cartridge was then analyzed first, while a new CG4+ cartridge was prepared, in order to minimize delays in obtaining blood gas measurements. The pH, pO_2_ and pCO_2_ data were temperature corrected for that turtle’s cloacal temperature, and iCa data were pH-corrected using previously described formulas (Keller *et al.*, 2012). HCO_3_ concentration was calculated with the Henderson–Hasselbalch equation, temperature-corrected pH and temperature-corrected pCO_2_, with αCO_2_ and p*K* values calculated via species-specific equations for Kemp’s ridley sea turtles (Stabenau and Heming, 1993). Three samples, two from the 6 h pool group and one from the 24 h pool group, did not produce iCa data due to failure of the i-STAT cartridge for iCa only. Thirty-nine of the 54 samples analyzed had undetectable lactate, i.e. below the limit of detection of 0.30 mmol/l, and were assigned a value of one-half the detectability limit (0.15 mmol/l) for statistical analysis of lactate data. Since the potential effect of delay in analysis time on pH and blood gas measurements in turtles has not been determined, we also report the time lag (min) between collection of the blood sample and analysis in the i-STAT machine, with the latter defined as the moment the machine first displayed ‘Calibrating.’

#### Complete blood counts (CBC)

Complete blood counts, including hematocrit, total WBC count and WBC differential count, were determined within 18 h for Sample 1 and within 36 h for Samples 2 and 3. Hematocrit was determined manually using standard capillary tube and centrifugation methods, while WBC was assessed using a hemocytometer and Phloxine B solution. Total leukocyte count was performed manually with a direct leukocyte counting method, while the differential WBC counts were performed by a single board-certified veterinary clinical pathologist (D. Davis); for full methodological details see Hunt *et al.* (2016a). Of the CBC data, only WBC, H/L ratio and hematocrit were analyzed statistically, since these three measures are known to be affected by transport in other species (Knowles and Warriss, 2007). WBC and H/L ratio typically increase due to the adrenal stress response and are known to increase in this species due to transport stress (Knowles and Warriss, 2007; Hunt *et al.*, 2016a), while hematocrit has been reported to increase due to transport-related dehydration in some other vertebrates (Knowles and Warriss, 2007). Since only limited hematologic data have been published for Kemp’s ridley turtles, mean ± SEM for other CBC variables are also reported here (see Results). One sample (Sample 2 from a turtle in the 24 h pool group) was found to be hemolyzed and did not produce any CBC data.

#### Corticosterone assay

Unextracted plasma samples were assayed for corticosterone using a double-antibody ^125^I radioimmunoassay previously validated for Kemp’s ridley turtle plasma (Hunt *et al.*, 2012; catalog #07-120103, MP Biomedicals, Solon, OH, USA). The manufacturer’s protocol was employed except that all samples, standards, and reagents were used at half-volume and an additional low standard was added to the standard curve (created by mixing equal volumes of assay buffer and the manufacturer’s lowest standard). To keep samples within the range of the standard curve, turtle plasma samples were diluted 10-fold in assay buffer, with final results then multiplied by 10. Intra- and inter-assay variations were both <10%. Non-specific binding tubes and blanks were assayed in quadruplicate, and standards, controls and samples were assayed in duplicate. Any samples with a coefficient of variation >10% between duplicate tubes, or that fell outside 10–90% bound, were rediluted and reassayed accordingly. For further assay details, including antibody cross-reactivities and sensitivity, see Hunt *et al.* (2012, 2016a).

### Data analysis

Variables were divided into two multivariate datasets for analysis (following Hunt *et al.*, 2016a): (1) four ‘stress-associated measures’ (corticosterone, glucose, WBC and H/L ratio), i.e. variables previously shown to increase significantly during ground transportation of this species, and (2) nine ‘clinical health measures’ (pH, pO_2_, pCO_2_, HCO_3_, sodium, potassium, ionized calcium, lactate and hematocrit), i.e. variables commonly used to monitor clinical health but that have not exhibited significant transport-associated changes in previous studies of this species. Lactate, corticosterone, WBC and H/L data were log-transformed before analysis to adjust for non-normal distributions based on skewness and kurtosis. All data were also inspected by a veterinarian (C. Innis) for any clinically relevant deviations from expected values for healthy individuals (as compared to data reported for juveniles of this species in Stabenau *et al.*, 1991; Moon *et al.*, 1997; Hoopes *et al.*, 2000; Innis *et al.*, 2007, 2009; Snoddy *et al.*, 2009; Keller *et al.*, 2012; Coleman *et al.*, 2016; Hunt *et al.*, 2016a; Stacy and Innis, 2017).

Descriptive statistics (mean ± SEM) were used to summarize the data set. To investigate the effect of transport and pool group (6 vs 24 h) on measured physiological variables, we used a multivariate generalized linear mixed model (GLMM) framework with normal probability distribution and identity link function. A mixed model allowed both fixed and random components to be fitted in the model. In this case, individual turtle was included as a random effect to account for individual-level variability, and fixed explanatory variables incorporated into the model were sampling time point (Sample 1 = pre-transport baseline; Sample 2 = immediately post-transport; Sample 3 = after pool), pool recovery duration (6 h or 24 h) and an interaction term (sampling time point × pool group). For each of the two multivariate datasets (stress-associated measures or clinical health measures), we conducted a series of GLMM analyses that each compared two sampling time points to investigate the influence of different stages of transport experienced by the turtles: (1) determine the effect of transport (Sample 1 pre-transport baseline vs. Sample 2 immediately post-transport); (2) examine whether time in a recovery pool altered or improved turtle physiological status and whether different pool time durations (6 or 24 h) had different effects (Sample 2 immediately post-transport vs Sample 3 after-pool) and (3) examine whether the pools allowed turtles to fully recover to a pre-transport baseline health state and whether different pool time durations (6 or 24 h) were associated with differing degrees of recovery (Sample 3 after-pool vs Sample 1 pre-transport baseline). In order to compare corticosterone changes to prior literature, individual trends in corticosterone were also summarized as percentage of turtles exhibiting an increase or decrease during transport (Sample 1 to same turtle’s Sample 2) or during pool recovery (Sample 2 to same turtle’s Sample 3), along with calculation of mean percentage change from the prior sample. Data were analyzed using the statistical software SPSS (v20.0.0 for Macintosh OSX) and Prism (v6.0g for Macintosh OSX) with alpha (significance threshold) set at 0.05.

## Results

### Effect of transport

**Figure 1:**
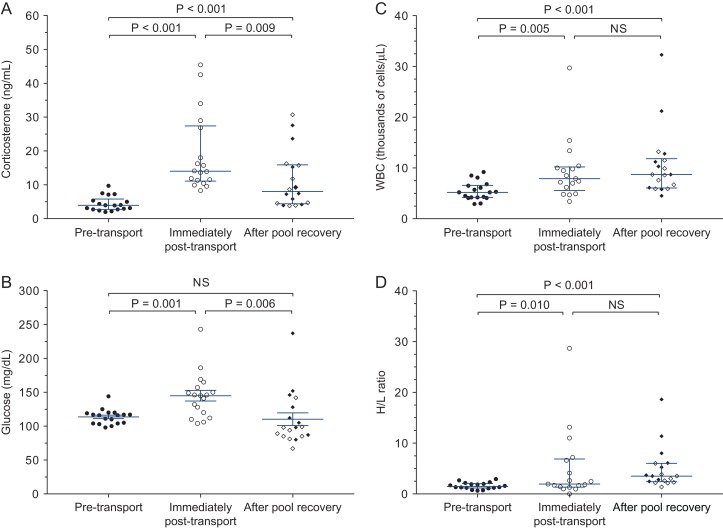
Plasma corticosterone (**A**), plasma glucose (**B**), total white blood cell count (**C**) and heterophil/lymphocyte ratio (**D**) of 18 juvenile Kemp’s ridley sea turtles transported out-of-water for ~24 h and then placed in saltwater pools for recovery. Symbols indicate data from individual turtles; central bar and whiskers indicates mean ± SEM for normally distributed data (glucose) or median ± interquartile interval for non-normal data (corticosterone, WBC and H/L ratio). ‘After pool recovery’ data includes turtles sampled after 6 h (closed diamonds) or 24 h (open diamonds) in pools. *P* values for individual variables shown only if multivariate GLMM analysis indicated an overall significant difference in that group of variables; ‘NS’ = no significant difference in the multivariate GLMM

Ground transportation of turtles from Massachusetts to Georgia affected all four stress-associated measures in Kemp’s ridley turtles. There was an overall significant difference between Sample 1 and Sample 2 (*F*_4,26_ = 16.00, *P* < 0.001), with significant increases in corticosterone (*F*_1,29_ = 64.51, *P* < 0.001), glucose (*F*_1,29_ = 14.26, *P* = 0.001), WBC (*F*_1,29_ = 9.13, *P* = 0.005) and H/L ratio (*F*_1,29_ = 7.53, *P* = 0.01). Individual data points for these four variables are presented in Fig. 1 with means summarized in Table 1. On an individual basis, 100% of turtles showed an increase in corticosterone during transport (between Samples 1 and 2), with an average of a 5-fold increase.

**Table 1:** Stress-related measures and clinical health measures

	Sample 1	Sample 2	Sample 3	*P* values		
	Pre-transport	Immediately post-transport	After pool (all turtles)	Samples 1 vs 2	Samples 2 vs 3	Samples 1 vs 3
Stress-associated measures						
Corticosterone (ng/ml)	4.41 ± 0.52	19.02 ± 2.72	11.4 ± 2.00	<0.001**	0.009**	<0.001**
Glucose (mg/dl)	113 ± 2.6	145 ± 7.9	110 ± 9.4	0.001**	0.006**	0.716
WBC count (cells/μl)	5.4 ± 0.43	9.4 ± 1.48	10.6 ± 1.57	0.005**	0.443	<0.001**
H/L ratio	1.6 ± 0.15	5.2 ± 1.72	4.9 ± 0.99	0.010**	0.292	<0.001**
Clinical health measures						
pH	7.52 ± 0.02	7.53 ± 0.01	7.53 ± 0.01	0.283	0.981	0.524
pO_2_ (mm Hg)	72.1 ± 2.05	68.7 ± 2.11	66.5 ± 2.16	0.479	0.378	0.043*
pCO_2_ (mm Hg)	40.2 ± 2.12	38.3 ± 0.65	38.3 ± 0.65	0.422	0.132	0.853
HCO_3_ (mmol/l)	37.6 ± 0.93	38.3 ± 0.76	38.5 ± 0.97	0.336	0.277	0.059
Na (mmol/l)	151.2 ± 0.50	151.3 ± 0.73	152.2 ± 0.58	0.062	0.415	0.149
K (mmol/l)	3.5 ± 0.14	3.0 ± 0.08	3.4 ± 0.08	0.004**	0.003**	0.581
iCa (mmol/l)	0.83 ± 0.02	0.81 ± 0.02	0.86 ± 0.02	0.803	0.094	0.190
Lactate (mmol/l)	0.62 ± 0.43	0.10 ± 0.06	0.65 ± 0.32	0.375	0.070	0.398
Hematocrit (%)	31.6 ± 0.47	32.5 ± 0.94	28.6 ± 0.55	0.555	0.004**	0.001**

Mean (±SEM) of four stress-associated variables and nine clinical health measures measured in 18 juvenile Kemp’s ridley sea turtles before transport (Sample 1), immediately after 24 h of ground transport (Sample 2) and after a variable period of time in a post-transport recovery pool (Sample 3; data shown here combine 6 h and 24 h pool groups). A significant difference (shown in bold with asterisks) between Samples 1 vs 2 represents effect of transport, between Samples 2 vs 3 represents effect of pool recovery and between Samples 1 and 3 represents failure to completely return to pre-transport baseline.

Transportation also influenced the clinical health measures (multivariate analysis of pH, pO_2_, pCO_2_, HCO_3_, sodium, potassium, iCa, lactate and hematocrit; *F*_9,19_ = 7.075, *P* < 0.001), with the overall significant difference largely attributable to a decline in potassium during transport (*F*_1,27_ = 9.73, *P* = 0.004).

### Pool time after transport

**Figure 2:**
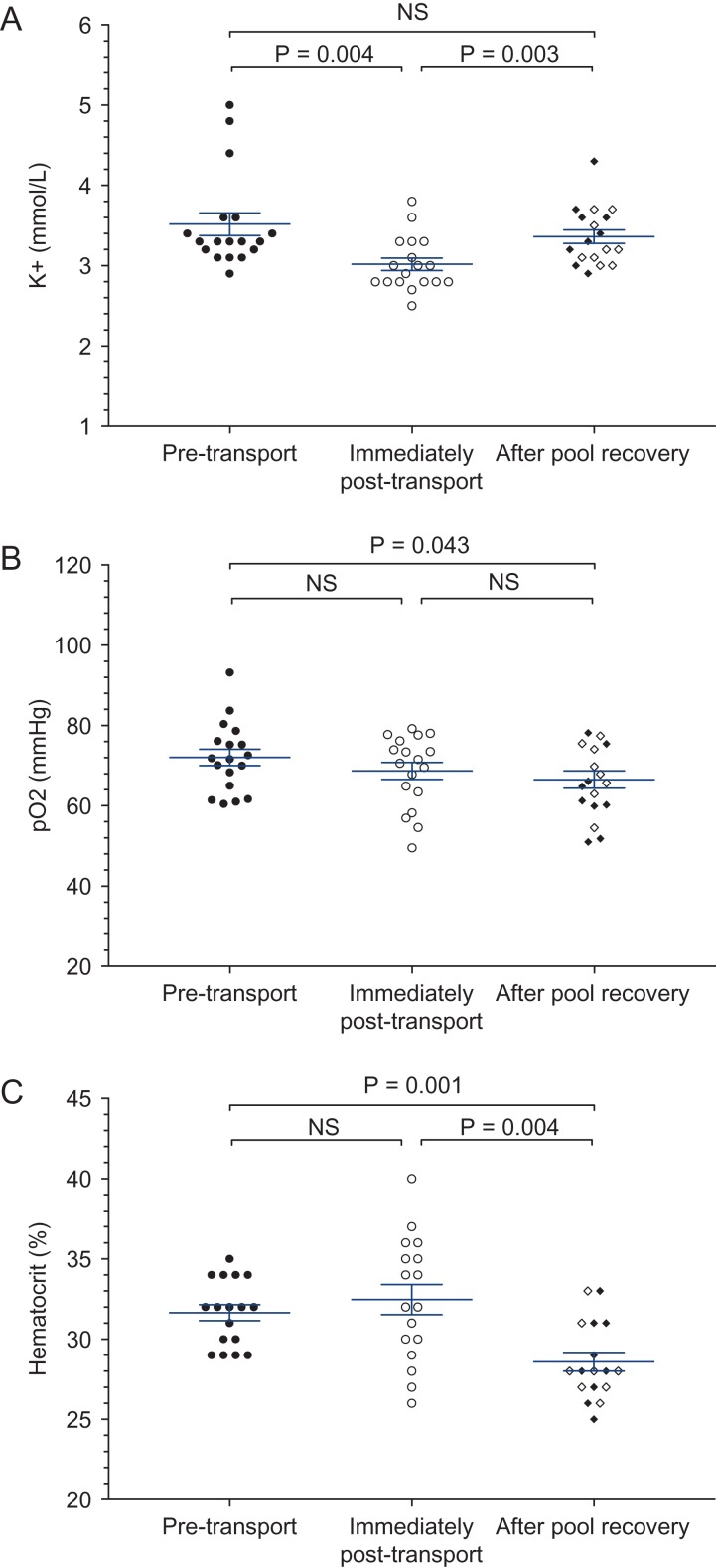
Potassium (**A**), pO_2_ (**B**) and hematocrit (**C**) of 18 juvenile Kemp’s ridley sea turtles transported out-of-water for ~24 h and then placed in saltwater pools for recovery. Symbols indicate data from individual turtles; central bar and whiskers indicates mean ± SEM. ‘After pool recovery’ data includes turtles sampled after 6 h (closed diamonds) or 24 h (open diamonds) in pools. *P* values for individual variables shown only if multivariate GLMM analysis indicated an overall significant difference in that group of variables; ‘NS’ = no significant difference in the multivariate GLMM

After time in the pools, multivariate analyses of stress-associated (*F*_4,26_ = 6.21, *P* = 0.001; Fig. 1) and clinical measures (*F*_9,16_ = 4.42, *P* = 0.005; Fig. 2) indicated significant improvements compared to immediately post-transport (Sample 2 vs Sample 3). Specifically, two of the four stress-associated measures declined significantly from their post-transport peaks, corticosterone (*F*_1,29_ = 7.74, *P* = 0.009) and glucose (*F*_1,29_ = 8.70, *P* = 0.006; Fig. 1 and Table 1), while potassium increased significantly from its post-transport nadir (*F*_1,24_ = 10.77, *P* = 0.003; Fig. 2 and Table 1). WBC and H/L ratio, however, remained elevated (Fig. 1 and Table 1), and hematocrit, which had not changed during transport, declined after time in the pools (*F*_1,24_ = 10.77, *P* = 0.004; Fig. 2 and Table 1). On an individual basis, corticosterone declined in 95% (17 of 18) of turtles during pool recovery, with on average a 2-fold decrease from Sample 2 to the same turtle’s Sample 3.

Comparing the two pool groups, multivariate analysis of the four stress-associated measures indicated improvements in the turtles given 24 h in the pools, as compared to turtles given only 6 h in the pools (*F*_4,26_ = 3.92, *P* = 0.13). This difference was driven largely by a significant reduction in H/L ratio in the 24 h group (*F*_1,29_ = 8.87, *P* = 0.006; Fig. 1 and Table 1). The nine clinical health measures did not significantly differ between 6 h and 24 h pool treatment groups (*F*_9,16_ = 1.67, *P* = 0.18).

Ultimately, even after a period of recovery in a pool (Sample 3), physiological parameters were still significantly different from baseline levels prior to transport (Sample 1) (stress-associated measures: *F*_4,28_ = 8.46, *P* < 0.001; clinical measures: *F*_9,18_ = 5.12, *P* = 0.002). Of the four stress-associated measures, glucose did return to pre-transport baseline levels (*P* = 0.72), even in turtles given only 6 h in the pools. Corticosterone, which had declined partially after 6 h in the pools, remained significantly elevated as compared to pre-transport baselines (*F*_1,31_ = 15.28, *P* < 0.001), even after 24 h in the pools. The two immune measures, WBC (*F*_1,31_ = 18.76, *P* < 0.001) and H/L ratio (*F*_1,31_ = 34.68, *P* < 0.001) also remained significantly elevated compared to pre-transport baselines. Potassium, the only one of the nine clinical health measures to show a significant change during transport, returned fully to pre-transport baselines (*F*_1,26_ = 0.312, *P* = 0.581). However, hematocrit (*F*_1,26_ = 14.18, *P* = 0.001) and pO2 (*F*_1,26_ = 4.52, *P* = 0.04) were both significantly lower in Sample 3 as compared to Sample 1. Throughout the study, turtles had exhibited small but sustained declines in both hematocrit and pO_2_ from Sample 1 to Sample 2 (*P* = 0.56 and 0.48, respectively) and again from Sample 2 to Sample 3 (*P* = 0.38 and 0.004, respectively), such that both variables ended up below pre-transport baselines (Fig. 2 and Table 1).

### Clinical condition

Pre-transport, immediately post-transport and post-pool physiologic data were generally within expected limits for healthy juveniles of this species, with the following exceptions. Two individuals (one in the 6 h pool group and one in the 24 h pool group) were outliers for their pre-transport data, with both affected by mild to moderate respiratory and metabolic acidosis and mild hyperkalemia (turtle # 14-647 [6 h pool group], pre-transport pH = 7.300, pO_2_ = 60.49 mm Hg, pCO_2_ = 72.1 mm Hg, HCO_3_ = 40.78 mmol/l, potassium = 4.8 mmol/l; turtle #14–732 [24 h pool group], pre-transport pH = 7.414, pO_2_ = 68.34 mm Hg, pCO_2_ = 49.8 mm Hg, HCO_3_ = 36.83 mmol/l, potassium = 5.0 mmol/l). Another individual (turtle # 14-473, 6 h pool group) was similarly characterized after 6 h of pool recovery (pH = 7.403, pO_2_ = 59.95 mm Hg, pCO_2_ = 49.6 mm Hg, HCO_3_ = 35.61 mmol/l, potassium = 4.3 mmol/l). One individual (turtle # 14-497, 6 h pool group) was affected by moderate hyperglycemia post-transport (Sample 2, glucose = 243 mg/dL), which persisted after the 6 h pool recovery (Sample 3, glucose = 237 mg/dL). For seven turtles, WBC was mildly to moderately increased (11 000–32 000 cells/μl) in the post-transport sample (Sample 2, three 6 h turtles) or post-pool recovery sample (Sample 3, four 6 h turtles—two of which also had high WBC in Sample 2—and two 24 h turtles).

#### Body mass, measurements, vital rates, sample timing and i-STAT timing

Mean body mass for turtles in this study (±SD) was 4.30 ± 0.94 kg, mean straight carapace length (notch to tip) was 30.18 ± 2.39 cm and straight carapace width (at widest point) was 28.05 ± 0.53 cm, with no significant differences between pool groups (*P *≫ 0.05 for all comparisons). Vital rates, additional CBC data and sample timing data are reported in Table 2. All vital rates remained within clinically acceptable ranges throughout the transport event, as judged by the attending veterinarians (C. Innis and T. Norton).

**Table 2:** Vital rates, hematologic data and timing parameters of eighteen juvenile Kemp’s ridley sea turtles assessed before transport (Sample 1), immediately after 24 h of ground transport (Sample 2) and after a variable period of time in a post-transport recovery pool (Sample 3; data shown here combine 6 and 24 h pool groups)

	Sample 1 (pre-transport)	Sample 2 (post-transport)	Sample 3 (after pool)
Vital rates (mean ± SEM)			
Temperature (°C)	25.21 ± 0.04	24.28 ± 0.10	24.88 ± 0.05
Heart rate (bpm)	47.00 ± 0.93	40.89 ± 1.07	42.61 ± 2.02
Respirations (per min)	5.11 ± 0.79	5.00 ± 0.60	4.54 ± 0.64
Hematologic data (mean ± SEM)			
Heterophils (%)	57.50 ± 2.50	66.00 ± 5.04	76.83 ± 2.20
Lymphocytes (%)	40.00 ± 2.33	26.44 ± 3.78	21.94 ± 2.23
Monocytes (%)	1.94 ± 0.41	0.94 ± 0.21	1.11 ± 0.21
Eosinophils (%)	0.56 ± 0.23	0.44 ± 0.18	0.11 ± 0.08
Heterophils (cells/μl)	3194 ± 327.6	6420 ± 1491.0	8588 ± 1558.5
Lymphocytes (cells/μl)	2111 ± 162.6	2109 ± 308.7	1877 ± 102.6
Monocytes (cells/μl)	106.8 ± 22.5	230.2 ± 147.7	129.3 ± 37.3
Eosinophils (cells/μl)	26.8 ± 10.7	51.3 ± 23.3	11.9 ± 8.3
Timing parameters (mean ± standard deviation)			
Bleed time (min)	1.94 ± 0.52	1.22 ± 0.61	2.10 ± 0.35
Handling time (min)	5.61 ± 1.22	4.26 ± 0.36	5.80 ± 0.97
CG4 lag time (min)	1.81 ± 1.07	1.46 ± 0.34	1.20 ± 0.20
CG8 lag time (min)	5.65 ± 1.42	5.00 ± 0.39	3.91 ± 1.5

Timing parameters are measured from time of first disturbance (time net placed in turtle's tank, or time turtle's transport crate was opened); 'Lag Time' for CG4 and CG8 represents delay between collection of blood sample and subsequent i-STAT analysis.

## Discussion

Kemp’s ridley turtles placed in unfamiliar saltwater pools after an overnight out-of-water ground transport demonstrated partial recovery from transport-related physiological stress. Of the five measures found to be significantly affected by transport, glucose and potassium normalized completely to pre-transport baselines after just 6 h in an unfamiliar saltwater pool. However, the other three variables did not all recover at the same rates. Corticosterone partially normalized by 6 h but then plateaued, remaining slightly elevated even after 24 h in the pools. H/L ratio was still at its post-transport peak after 6 h in the pool, but began to recover toward normal after 24 h in the pool. WBC count was the only stress-associated variable to show no recovery, remaining at or near its post-transport peak even after 24 h in a pool.

### Corticosterone

Mean corticosterone quintupled during transport, from ~4 to ~19 ng/ml. Before transport, no turtles had corticosterone >10 ng/ml; immediately after transport, all but one turtle had corticosterone >10 ng/ml, with five samples >20 ng/ml. Generally, the amplitude of this increase was similar to that seen after 26 h transport (from 2 to 12 ng/ml) in our earlier study of juvenile Kemp’s ridley turtles (Hunt *et al.*, 2012) and is greater than the minor, typically 2-fold, increase that is attributable to repeated handling and sampling in the absence of transport (Hunt *et al.*, 2016b). Other comparison points from sea turtle stress literature include 3- to 4-fold increases in corticosterone after single stressors such as 2d of osmotic stress, brief turning stress (turtle placed on its back), capture, or stranding (Kemp’s ridley turtles, Ortiz *et al.*, 2000; Gregory and Schmid, 2001; green turtles, Aguirre *et al.*, 1995; leatherback turtles, *Dermochelys coriacea*, Hunt *et al.*, 2016b). Blanvillain *et al.* (2008) reported a 7-fold increase in loggerhead turtles after capture and 20 h of out-of-water holding. Approximately 10- to 15-fold increases have been documented in several species after prolonged turning stress or combinations of several stressors (hawksbills, *Eretmochelys imbricata*, Jessop *et al.*, 2004; olive ridley turtles, *Lepidochelys olivacea*, Valverde *et al.*, 1999; loggerheads, Gregory and Schmid, 2001; green turtles, Jessop *et al.*, 2000). These varied stressors were not reported to cause acute mortality in the aforementioned studies, but follow-up after release is typically not performed and hence long-term sequelae are usually unknown. Finally, 20- to 30-fold increases in corticosterone have been documented at the NEAq clinic in newly admitted juvenile Kemp’s ridley turtles after a combination of cold-stunning, stranding and transport, resulting in mean corticosterone of ~40 ng/ml (Hunt *et al.*, 2012), with some associated mortality (Innis *et al.*, 2007, 2009, 2014). In short, the quintupling of corticosterone seen here after transport likely represents a moderate but not a maximal stress response.

Corticosterone showed partial recovery (decline from post-transport peak) in almost all (95%) turtles after even a short period in a saltwater pool. Several turtles still retained high circulating corticosterone >10 ng/ml even after 24 h in the pool, but these individuals were those that also had the highest Sample 2 concentrations; that is, their Sample 3 corticosterone concentrations, though relatively high compared to other turtles, represent a decline from that individual turtle’s Sample 2. There was no further decline in corticosterone with more time in the pool (no difference between 6 and 24 h pool groups), possibly indicating that the novel environment of the unfamiliar pool was itself causing a lingering, though more minor, stress response.

### Glucose

Elevations in glucose are one of the most consistent physiological responses to transport stress in vertebrates (Knowles and Warriss, 2007; Becerril-Herrera *et al.*, 2010; do Carmo *et al.*, 2013; Vasquez-Galindo *et al.*, 2013). This is presumably a consequence of the adrenal stress response, which induces a variety of metabolic changes (increased gluconeogenesis, alterations in insulin response, etc.) to increase fuel availability for dealing with whatever stressor the animal may be facing (Mizock, 1995; Mormede *et al.*, 2007). As expected, glucose exhibited a strong and significant elevation immediately post-transport, to concentrations very similar to those previously reported for transport stress in this species (Hunt *et al.*, 2016a). However, placement in pools resulted in a rapid and significant decline in glucose, which reached baseline after even just 6 h in the pools.

### Potassium

Similarly, potassium, which had declined mildly but significantly during transport, returned rapidly to baseline after just 6 h in the pools. Potassium concentrations were not affected in our previous study on transport stress in this species (Hunt *et al.*, 2016a), and turtles in the present study maintained normal potassium concentrations despite the statistically detected decrease. Hypokalemia has been reported in other vertebrates after transportation (Rock *et al.*, 1990; Becerril-Herrera *et al.*, 2010; Vasquez-Galindo *et al.*, 2013). Generally, the vertebrate stress response tends to involve increases not only of corticosterone but also, to some degree, the related adrenal hormone aldosterone, which can reduce plasma potassium concentrations (Guyton and Hall, 2000). Other factors that may have reduced plasma potassium concentrations during transport include fasting, absence of sea water ingestion, diuresis induced by fluid therapy, or fluid loss from the gastrointestinal tract (e.g. undetected vomiting).

### Immune measures

One of the most consistent effects of the adrenal stress response in vertebrates is a redistribution of immune cells (Martin, 2009; Johnstone *et al.*, 2012). Classically, corticosterone induces certain classes of WBC to shift from blood plasma to interstitial compartments, as if ‘deployed’ to peripheral tissues as a defense against potential new wounds. This phenomenon often causes an increase in H/L ratio, and there may be an overall elevation of total WBC count as well (Martin, 2009; Johnstone *et al.*, 2012). In this study, the two immune measures WBC and H/L ratio both elevated during transport as expected, and interestingly these two measures proved most resistant to recovery. WBC remained strongly elevated in both pool groups, with no apparent effect of any duration in pools, while H/L ratio showed partial recovery only in the 24 h pool group. The Georgia pools were a novel environment—pool dimensions, wall color, acoustic environment, water temperature and the visible above-water environment were all, unavoidably, slightly different than the tanks in Massachusetts. Given the novelty of the pools and the continued elevation in corticosterone, it is perhaps unsurprising that turtles would continue to exhibit some physiologic indications of stress.

### Other clinical health measures

Most of the remaining clinical health measures—pCO_2_, pH, bicarbonate, lactate, iCa, sodium—exhibited no significant changes at any point in the study. There were minor, yet statistically significant, decreases in pO2 and hematocrit, but in our judgment these changes were not clinically meaningful. pO_2_ was likely affected by variable activity and respiratory frequency prior to sampling (i.e. turtles may have been breathing less frequently while resting during pool recovery; K. Hunt and A. Kennedy, pers. obs.). The minimal decrease in hematocrit could have been the result of fluid therapy, or increased water ingestion upon entry to the recovery pools.

### Clinical condition of individual turtles

Despite the stress of transport, health measures for the majority of individuals remained within clinically acceptable limits. There are no well-established reference intervals for Kemp’s ridley sea turtle blood data; however, a number of studies provide comparative data for presumed healthy wild or captive rehabilitated Kemp’s ridley sea turtles (Stabenau *et al.*, 1991; Moon *et al.*, 1997; Hoopes *et al.*, 2000; Innis *et al.*, 2007, 2009; Snoddy *et al.*, 2009; Keller *et al.*, 2012; Coleman *et al.*, 2016; Hunt *et al.*, 2016a; Stacy and Innis, 2017). Health data in the present study were generally consistent with these previous studies. One individual was affected by moderate hyperglycemia after transport, which persisted after six hours of pool recovery. Seven individuals were affected by mild to moderate leukocytosis after transport and/or pool recovery. ‘Stress hyperglycemia’ and the ‘stress leukogram’ are widely recognized physiologic responses in vertebrates and have been previously described in hospitalized Kemp’s ridley turtles (Innis *et al.*, 2009; Keller *et al.*, 2012). While these conditions often resolve spontaneously during hospitalization (Innis *et al.*, 2017; Stacy and Innis, 2017), the effect of these conditions on post-release outcome is unknown.

One turtle was affected by mild respiratory and metabolic acidosis and slight hyperkalemia after pool recovery, similar to that seen in two turtles prior to transport. Given that these individuals were judged to be otherwise healthy, it is likely that these findings represent variability within the normal physiologic repertoire of this species. That is, these turtles may have been sampled after a period of submergence, during which such physiologic changes are known to occur (Stabenau *et al.*, 1991; Hoopes *et al.*, 2000; Innis *et al.*, 2010, 2014). Anecdotally, turtles observed after pool recovery were noted to be unusually quiet compared to typical behavior seen daily at the rehabilitation clinic in Massachusetts. Specifically, just before collecting Sample 3, most turtles were noted to be resting at the bottom of the pools with eyes closed (possibly asleep) and few were actively swimming (K. Hunt and A. Kennedy, pers. obs.). This may indicate an effect of fatigue caused by transport and, thus, may have resulted in turtles being sampled after an unusually prolonged period of submergence. It is possible, in fact, that the generally beneficial physiological effects of time in the pools may be partly attributable to resting or sleeping, i.e. recovery from fatigue.

### What is the optimal release phenotype for sea turtles?

Our study design assumes that return to pre-transport physiological baselines is desirable, but it is possible that the ‘optimal release phenotype’ for newly released sea turtles may not be identical to pre-transport baselines taken at rest. For example, it is conceivable that mild elevations in corticosterone upon release may not be detrimental or may even be beneficial. In many species, corticosterone promotes ‘escape’ or migratory behaviors as well as promoting foraging, such that stressed individuals with higher corticosterone tend to forage more widely and may not return to familiar areas (in birds, abandonment of territories; Breuner and Hahn, 2003; Wingfield *et al.*, 2015). Movement patterns and residency times of juvenile sea turtles are largely unknown at present, but it is possible that mild elevations in corticosterone could stimulate greater locomotion, greater foraging and alterations in spatial movement generally, which are not necessarily negative outcomes. Further research employing satellite tags would be necessary for exploring normal behavior of never-stranded turtles as compared to the behavior and spatial movement of turtles released in various physiological states after transport.

## Conclusions

In sum, a 6 h period in a saltwater pool after ground transport was generally effective at normalizing several transport-associated physiologic changes, while additional time up to 24 h had only mild additional effects. Though pool recovery regimes tested did not normalize all measures, the pronounced recovery of some parameters, particularly glucose, potassium and to some extent corticosterone, is encouraging. Our interpretation is that a ‘soft release’ method of holding sea turtles briefly in saltwater pools near release beaches can help reduce the physiological changes that occur due to the stress of transport. Further, the ‘soft release’ period can be relatively short; a 6 h ‘same-evening release’ approach had almost the same effects as the more logistically complex 24 h ‘next-morning release’ approach. We suggest that soft releases are not essential, since turtles remained in good clinical condition after the overnight transport. However, in situations where saltwater pools are readily available close to the release site, pool recovery may be a practical and beneficial step for preparing turtles for release to the sea.
